# Cyclo-oxygenase 2, a putative mediator of vessel remodeling, is expressed in the brain AVM vessels and associates with inflammation

**DOI:** 10.1007/s00701-021-04895-z

**Published:** 2021-06-29

**Authors:** Sara Keränen, Santeri Suutarinen, Rahul Mallick, Johanna P. Laakkonen, Diana Guo, Ludmila Pawlikowska, Behnam Rezai Jahromi, Tuomas Rauramaa, Seppo Ylä-Herttuala, Doug Marchuk, Timo Krings, Timo Koivisto, Michael Lawton, Ivan Radovanovic, Helen Kim, Marie E. Faughnan, Juhana Frösen

**Affiliations:** 1 Hemorrhagic Brain Pathology Research Group, NeuroCenter, Kuopio University Hospital, Kuopio, Finland; 2 A.I. Virtanen Institute for Molecular Sciences, University of Eastern Finland, Joensuu, Kuopio Finland; 3 Center for Cerebrovascular Research, Dept of Anesthesiology and Perioperative Care, UCSF, San Francisco, CA USA; 4 Department of Pathology, Kuopio University Hospital, University of Eastern Finland, Kuopio, Finland; 5 Division of Human Genetics, Duke University School of Medicine, Durham, NC USA; 6 Department of Neuroradiology, University Hospital Network Toronto, Toronto, Canada; 7 Department of Neurosurgery, NeuroCenter, Kuopio University Hospital, Kuopio, Finland; 8 Department of Neurosurgery, Barrow Brain and Spine Institute, Phoenix, AZ USA; 9 Department of Neurosurgery, University Hospital Network Toronto, Toronto, Canada; 10 Toronto HHT Centre, St. Michael’s Hospital and Li Ka Shing Knowledge Institute, Toronto, Canada; 11 Division of Respirology, Department of Medicine, University of Toronto, Toronto, Canada; 12 Department of Neurosurgery, Tampere University Hospital and University of Tampere, Elämänaukio 2, PoBox 33521, Tampere, Finland

**Keywords:** Brain arteriovenous malformation, Inflammation, Cyclo-oxygenase-2, Vessel remodeling, Intracranial hemorrhage, Non-steroidal anti-inflammatory drugs

## Abstract

**Background:**

Brain arteriovenous malformations (bAVM) may rupture causing disability or death. BAVM vessels are characterized by abnormally high flow that in general triggers expansive vessel remodeling mediated by cyclo-oxygenase-2 (COX2), the target of non-steroidal anti-inflammatory drugs. We investigated whether COX2 is expressed in bAVMs and whether it associates with inflammation and haemorrhage in these lesions.

**Methods:**

Tissue was obtained from surgery of 139 bAVMs and 21 normal Circle of Willis samples. The samples were studied with immunohistochemistry and real-time quantitative polymerase chain reaction (RT-PCR). Clinical data was collected from patient records.

**Results:**

COX2 expression was found in 78% (109/139) of the bAVMs and localized to the vessels’ lumen or medial layer in 70% (95/135) of the bAVMs. Receptors for prostaglandin E2, a COX2-derived mediator of vascular remodeling, were found in the endothelial and smooth muscle cells and perivascular inflammatory cells of bAVMs. COX2 was expressed by infiltrating inflammatory cells and correlated with the extent of inflammation (*r* = .231, *p* = .007, Spearman rank correlation). COX2 expression did not associate with haemorrhage.

**Conclusion:**

COX2 is induced in bAVMs, and possibly participates in the regulation of vessel wall remodelling and ongoing inflammation. Role of COX2 signalling in the pathobiology and clinical course of bAVMs merits further studies.

**Supplementary Information:**

The online version contains supplementary material available at 10.1007/s00701-021-04895-z.

## Introduction

Arteriovenous malformations of the brain (bAVM) are rare vascular anomalies that may bleed causing epilepsy, neurological deficits, or even death [2]. BAVMs form due to dysregulated angiogenesis [13, 22]. Although there are multiple genes and signaling pathways in which mutations can cause dysregulation of angiogenesis leading to bAVM formation [13], recent reports demonstrate that in the majority of sporadic bAVMs, the presence of activating somatic KRAS mutations in endothelial cells can explain the formation of the bAVM [19, 22, 23].

Once formed, bAVMs may remain stable, enlarge, or rupture. Enlargement of the bAVM means growth of new vessels (angiogenetic proliferation) and/or dilatation of existing ones (ectatic remodeling). Rupture may occur as fulminant hemorrhage that has a fatality of approx. 25% [25], or as a subclinical or minor hemorrhage detected in MRI as hemosiderin accumulation [1, 29] and possibly causing epilepsy or headache rather [1] than neurological deficits. While the fulminant hemorrhage is thought to most often occur from ectatic dilatations of draining veins [16] or from intranidal aneurysms [21] arising from dilated nidal vessels, the minor hemorrhages seem to result rather from rupture of smaller nidal vessels [9]. These nidal bAVM vessels are pathologically enlarged capillaries pathognomonic to bAVMs [30] and form the arteriovenous shunts that lead to the abnormally high flow characteristic to bAVM vessels [25]. How the nidal vessels as well as the draining veins remodel under these abnormally high flow and pressure conditions eventually determines the clinical course of the bAVM.

High flow induces dilation and eventually structural enlargement of the vessel lumen as a physiological response that ensures sufficient blood flow through the vessels. This physiological adaptation is mediated by endothelial cells (EC) that are sensitive to the shear stress produced by blood flow [4, 7, 25, 28]. In addition to the physiological dilatative (ectatic) remodeling of the vessel wall, focal high shear stress caused by very high flow can trigger formation of aneurysms [12]. These pathological dilations of the vessel are characterized by abnormal wall structure that may rupture and often become inflamed [8, 12, 14]. Cyclo-oxygenase 2 (COX2) is an enzyme that produces mediators of this inflammation and has a critical role in the formation and progression of aneurysms under high flow conditions [3, 12, 24]. The expression of COX2 is induced in ECs by high flow [28], which also induces in ECs the expression of pro-inflammatory chemokines recruiting inflammatory cells to the vessel wall [3, 4, 12].

Since in many bAVMS vessels are exposed to unusually high flow, we hypothesized that COX2 expression could be induced in them. This could lead to an enhanced inflammatory response through the COX2-PGE2-EP2-NFkB-COX2 autocrine feedback loop in inflammatory cells, as well as to ectatic vessel remodeling of these bAVM vessels, possibly somewhat similarly as in the formation and progression of intracranial aneurysms (IA). Nidal vessels of bAVMs are indeed known to develop aneurysms (Online resource, Fig. S1), and when present, these intranidal aneurysms are often the most likely site of hemorrhage [9]. Aneurysms may also form on the arteries feeding the bAVM (Online resource, Fig. S1). Moreover, pathological dilatations may form in the draining veins (Online resource, Fig. S1), and these venous ectasias can rupture as well [9]. In addition to these, mechanisms related to flow induced COX2-mediated vessel remodeling and through which bAVMs may evolve to rupture-prone ones and, COX2 also induces and amplifies inflammation [24]. Inflammatory cell infiltration has been associated with bAVM rupture [10], and the exact mechanism remains unclear which needs to be fully elucidated, the proteolytic injury caused to the extracellular matrix of the fragile bAVM vessels by the proteases secreted from infiltrating inflammatory cells could make the bAVM more rupture prone [10].

We investigated tissue samples of surgically treated human bAVMs whether COX2 expression is induced in bAVM vessels and whether it is associated with wall remodeling, inflammation, and signs of hemorrhage.

## Methods

### Tissue collection and patient data

A total of 139 bAVM tissue samples were collected from surgically treated patients at the Kuopio University Hospital (KUH, Kuopio, Finland), Toronto Western Hospital (TWH, Toronto, Canada), and at the University of California San Fransisco (UCSF, San Francisco, CA, USA). Autopsy-derived normal Circle of Willis samples (*n* = 21, from KUH) as well as bowel samples were used as controls. Clinical information was collected from patient records and included, e.g., age, sex, bAVM rupture status, and epileptic symptoms. In addition, prior micro-/macrohemorrhage was determined by presence of hemosiderin in the histological sections. All patients had sporadic bAVMs. A summary of patient demographics and the clinical presentation is given in Table 1.

**Table 1:** Association of patient demographics and clinical presentation with cyclo-oxygenase 2 expression

Clinical variable	KUH (62 samples)		*P*value	UCSF (49 samples)		*P* value	TWH (28 samples)		*P* value
	COX2 expression			COX2 expression			COX2 expression		
	+ (*n* = 49)	− (*n* = 13)		+ (*n* = 40)	− (*n* = 9)		+ (*n* = 20)	− (*n* = 8)	
Age (median and range)	41 years (12–67)	26 years (4–67)	.169	26 years (6–61)	31 years (13–66)	.270	37 years (17–75)	29 years (21–59)	.990
Sex (percentage of females)	53.7% (22/41)	54.5% (6/11)	1.000	35.2% (13/37)	66.7% (6/9)	.133	50.0% (10/20)	62.5% (5/8)	.686
Epilepsy	47.2% (17/36)	55.6% (5/9)	.722	0% (0/40)	0% (0/9)	–	–	–	–
Prior rupture	53.7% (22/41)	81.8% (9/11)	.165	54.1% (20/37)	33.3% (3/9)	.459	45.0% (9/20)	50.0% (4/8)	1.000
Embolization	73.2% (30/41)	45.5% (5/11)	.145	52.5% (21/40)	55.6% (5/9)	1.000	–	–	–
Radiosurgery	7.3% (3/41)	27.3% (3/11)	.101	2.5% (1/40)	0% (0/9)	1.000	–	–	–

### Immunohistochemistry and histological analysis

Histological sections were cut from formalin-fixed, paraffin-embedded tissue samples and stained with immunohistochemistry and immunofluorescence staining as described in detail in the Supplementary data. The detected COX2 expression, as well as the degree of inflammation and other histological variables, were scored semi-quantitatively, as described in details in the Electronic supplementary material.

### Real-time quantitative polymerase chain reaction

To confirm the expression of COX2 in bAVM tissues, we quantified COX2 expression with real-time quantitative polymerase chain reaction (RT-PCR) from 6 paraffin-embedded bAVM samples and 6 samples from normal Circle of Willis, as described in the Supplementary material.

### Statistical analysis

For nominal and ordinal variables, percentages and proportions are given and Chi-square test used for statistical comparison. For continuous variables, median and range are given and the two-tailed non-parametric Mann–Whitney *U* test and Spearman rank correlation test used for statistical comparison because the data deviated from normal distribution. Logistic regression was used for multivariate analysis. Statistics were calculated with SPSS 26.0 software (IBM Corp., Armonk, NY, USA) and alpha level was 0.05.

### Data Availability

Data can be made available in a pseudonymized format based on a written request and scientifically sound research plan approved by the principal investigator and fulfilling all the data handling and protection requirements of all the institutions involved.

## Results

### COX2 expression is induced in bAVM vessels

**Fig. 1:**
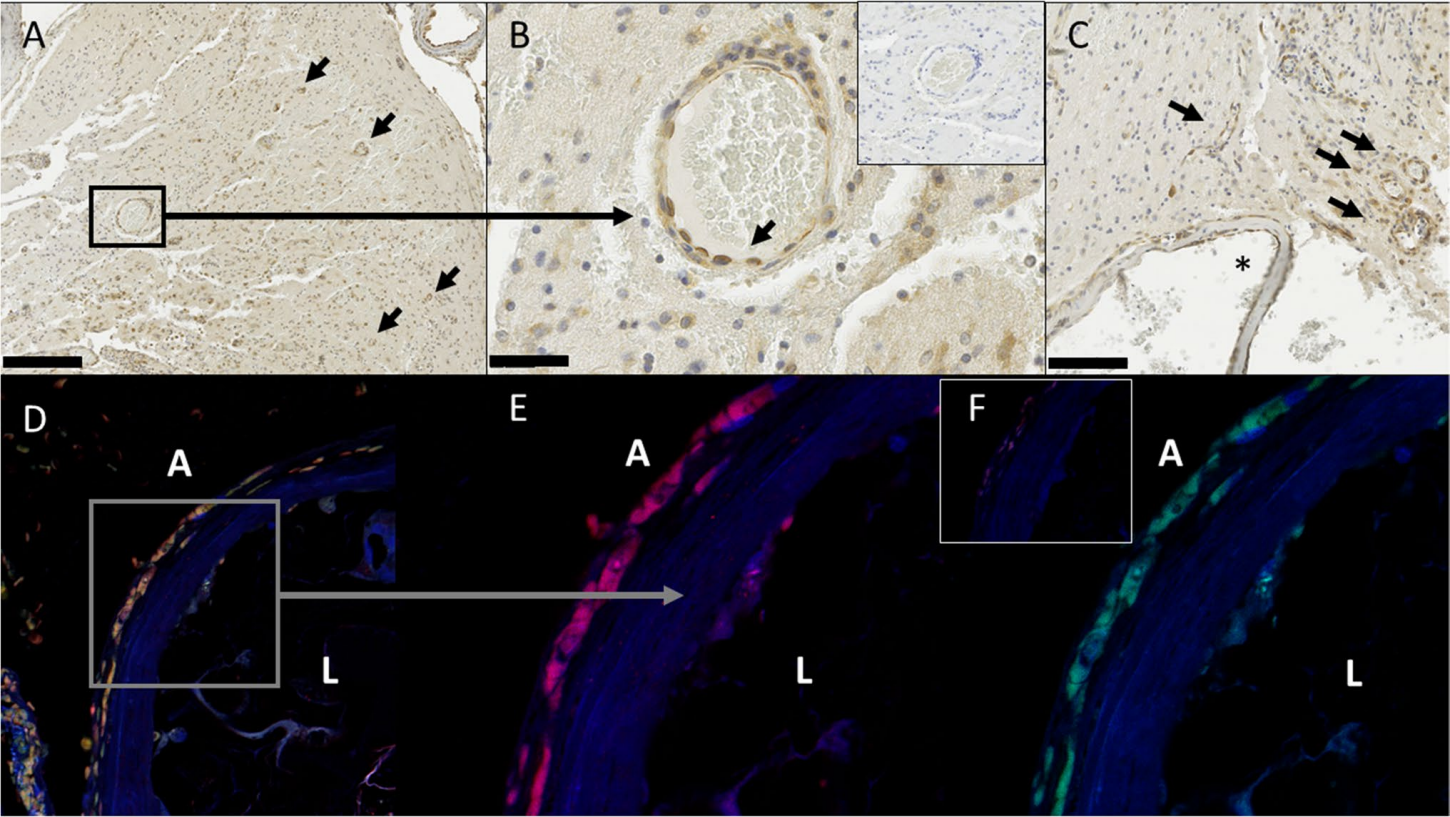
COX2 is expressed in the endothelial cells of bAVMs. The figure shows the expression of COX2 (in brown) in the endothelial cells of multiple small bAVM vessels (marked by black arrows) in a lower magnification view (**A**, scale bar 250 µm) and in a higher magnification close-up (**B**, scale bar 25 µm. Negative control from the same region is shown in the insert at the right upper corner of B). Another example of a lower magnification view with multiple COX2 expressing small bAVM vessels (marked by black arrows) is given in (**C**) (scale bar 100 µm), in which also an adjacent larger bAVM vessel (marked by an asterisk) shows COX2 expression (in brown) on the luminal and adventitial surface. Immunofluorescence double staining (**D**–**F**) show that COX2 is expressed in luminal and adventitial cells positive for CD31, supporting the endothelial (luminal double-positive cells) and inflammatory cell (adventitial double-positive cells) phenotype. Double-positive cells are visible as yellow in the overlay image (**D**), whereas COX2 is shown in red in the corresponding single channel image (**E**) and CD31 in green (**F**). The negative control section imaged with the same excitation and image acquisition settings as for (**D**–**F**) is shown in the upper corner of (**F**). The markings A and L in the microphotographs stand for adventitia and lumen, respectively

**Fig. 2:**
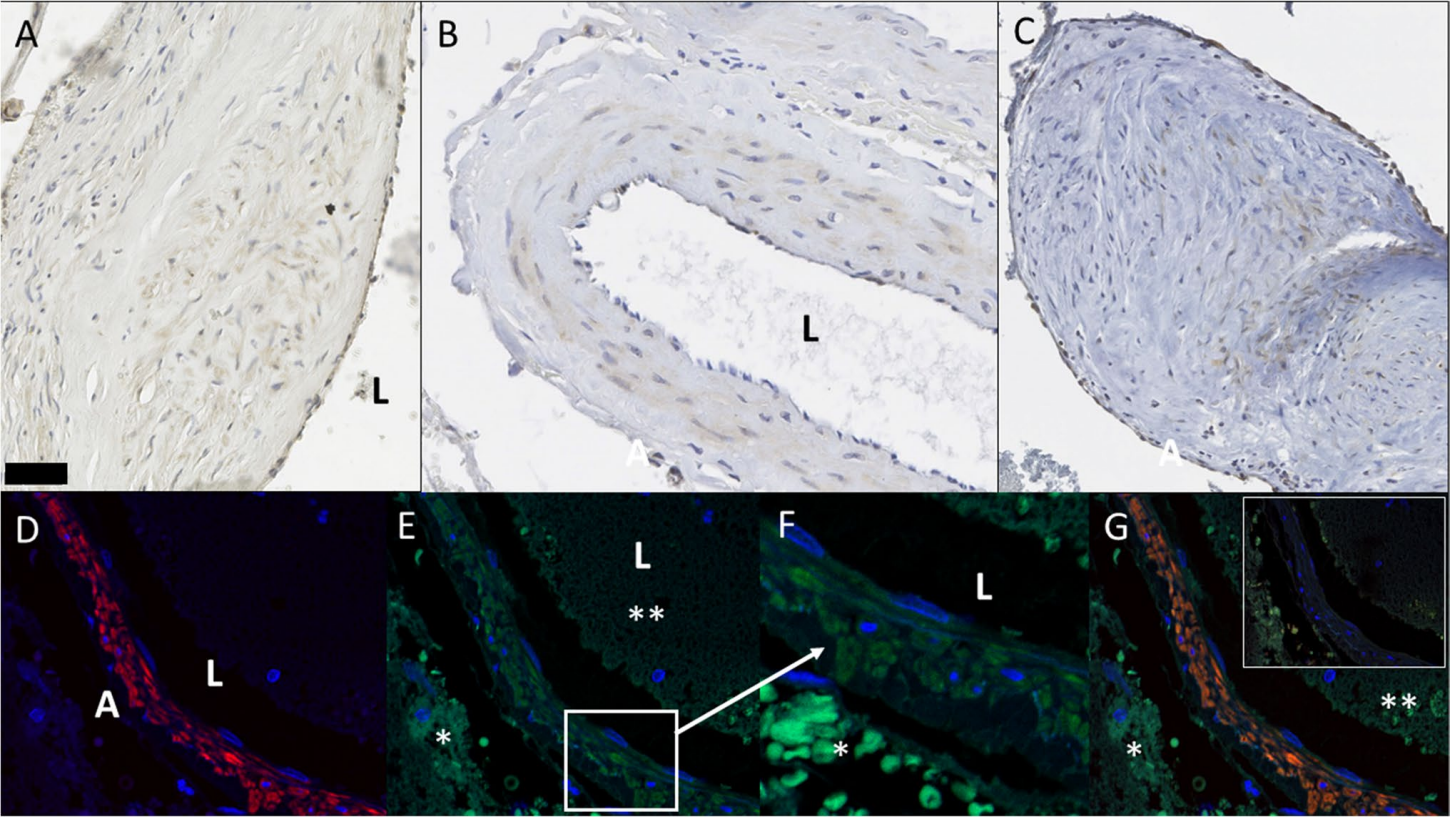
COX2 is expressed in the smooth muscle cells of bAVMs. The figure demonstrates COX2 expression (in brown) in the smooth muscle cell layer of large- and middle-sized bAVM vessels (**A**–**C**, scale bar 50 µm). Immunofluorescence double stainings (**D**–**G**) show in a lower magnification view (**D**, **E**) and in a higher magnification view (**F**, **G**) the wall of a small bAVM vessel with alfa smooth muscle actin-positive cells (in red in (**D**)) that show COX2 expression (in green in (**E**, **F**)). Double-positive cells are visible as yellow in the overlay image (**G**). An asterisk (*) shows red blood cells, which autofluorescate in all channels, also in the negative control. A double asterisk (**) marks vessel lumen. The negative control section imaged with the same excitation and image acquisition settings as for (**D**) and (**G**) is shown in the upper corner of (**G**). The markings A and L in the microphotographs stand for adventitia and lumen, respectively

Of the studied bAVM samples, 78% (109/139) expressed COX2 (Table 2; Figs.1 and 2). There was high regional variation in COX2 expression within samples with 48% (26/54) of the samples with stainings from multiple depths showing variation in COX2 score at different sites (Table 2). COX2 expression was not detected in control circle of Willis (*n* = 21). In 70% (95/135) of the bAVMs, COX2 was expressed in vessels (Table 2; Figs.1 and 2) regardless of the vessel size. COX2 was mostly seen in luminal surfaces and also in SMCs of some vessels (Figs. 1 and 2).

**Table 2:** Phenotype of cyclo-oxygenase 2 (COX2)-expressing cells as defined according to their morphological presentation in the immunostainings for COX2

Phenotype of cyclo-oxygenase 2 expressing cells	Percentage of + samples	Intrasample consensus
Overall in the tissue sample	78.4% (109/139)	48.1% (26/54)
Endothelial cells	50.4% (68/135)	88.1% (37/42)
Vascular smooth muscle cells	20.0% (27/135)	83.3% (35/42)
Inflammatory cells	75.6% (102/135)	97.6% (40/41)
Parenchymal cells (glial cells and neurons)	39.3% (53/135)	80.5% (33/41)

Phenotype of COX2 expressing cells as well as regional variation in the extent of COX2 expression was observed in most of the samples showing COX2 expression. The consensus presented in this table is defined as the percentage of samples that obtained a same COX2 expression score in sections cut from different regions of the sample

COX2 expression was found in 4 of the 6 bAVM samples studied with RT-PCR, but COX2 expression in Circle of Willis samples was undetermined (Supplemental data, Fig. S2).

### COX2-mediated signaling and bAVM vessel remodeling

**Fig. 3:**
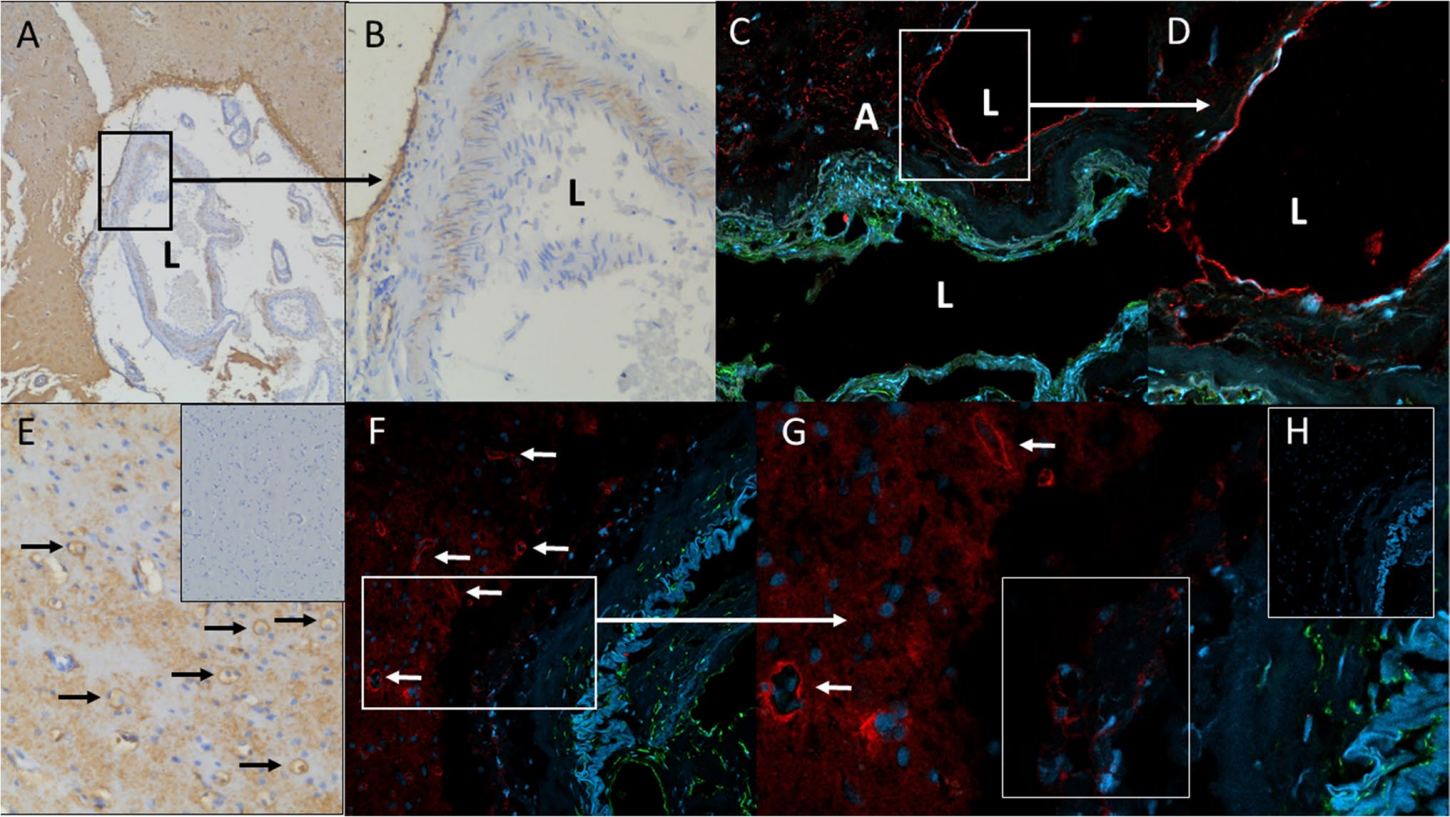
EP2, receptor for prostaglandin E2 is expressed in bAVMs. The figure shows EP2 expression (in brown) in smooth muscle cells of bAVM vessel in a lower magnification view (**A**) and in a higher magnification view (**B**). Immunofluorescence double staining (alfa smooth muscle actin in green and EP2 in red) in a lower magnification view (**C**) and in a higher magnification view (**D**) demonstrate EP2 expression in luminal cells of a large bAVM vessel. Based on their morphology these cells are endothelial cells (**C**, **D**). EP2 expression was also observed in the endothelium of smaller bAVM vessels (in brown in (**E**), and same area in negative control from adjacent section is presented in the upper corner of (**E**)). Immunofluorescence double staining in a lower magnification view (**F**) and in a higher magnification view (**G**) also demonstrates EP2 expression (in red) in the endothelium of small bAVM vessels (**F**). In addition, EP2 expression was observed in inflammatory cells (adventitial cells negative for alfa smooth muscle actin in green and with a mononuclear morphology) (**G**). The negative control section (**H**) imaged with the same excitation and image acquisition settings as for (**F**) and (**G**) is shown in the upper corner of (**G**). The markings A and L in the microphotographs stand for adventitia and lumen, respectively

**Fig. 4:**
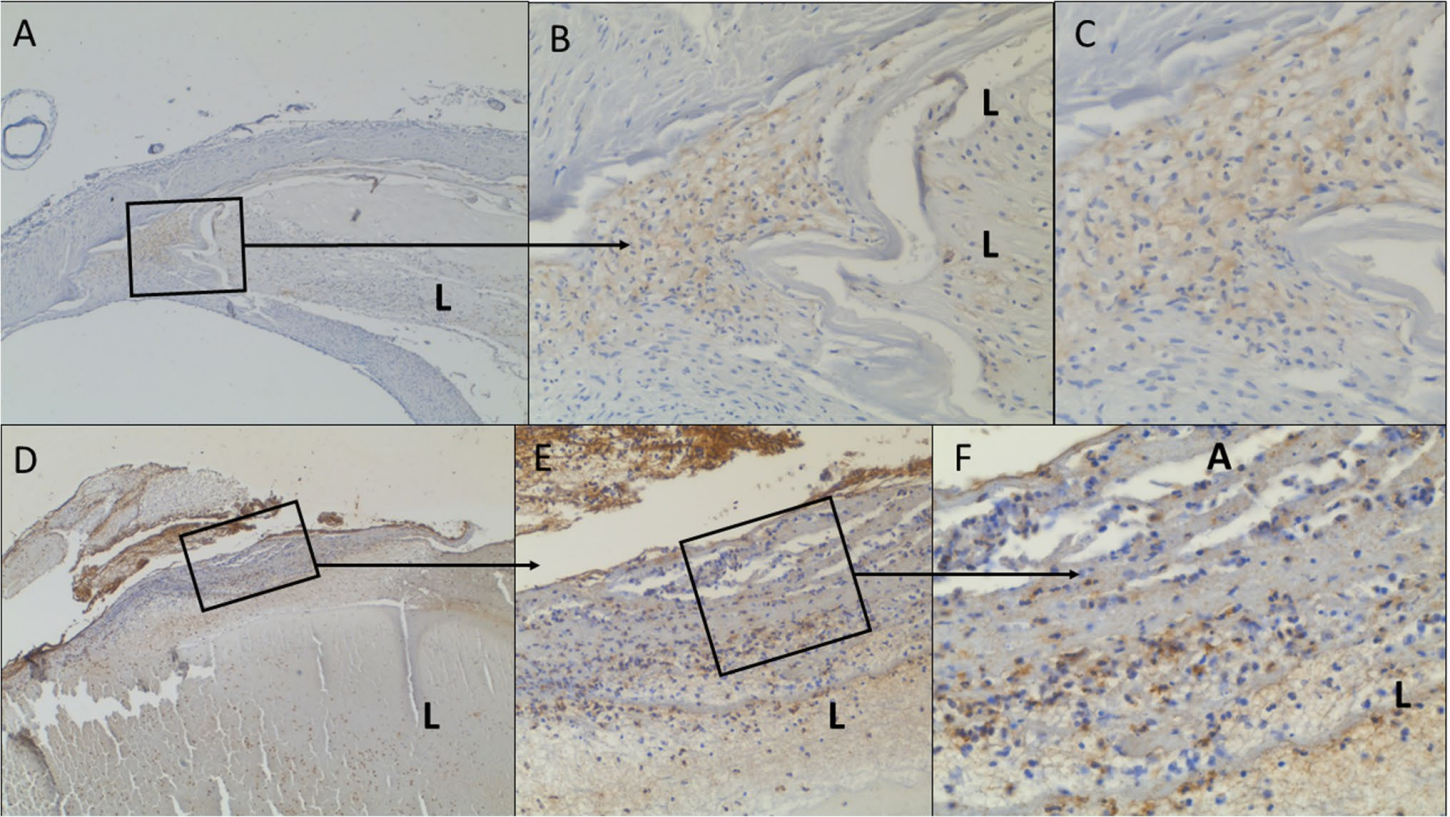
EP2 is expressed in bAVM vessels undergoing remodeling. The figure shows EP2 expression (in brown) in lower magnification views (**A**, **D**) and in higher magnification views (**B**, **C** and **E**, **F**) in adaptive (upper pictures, site of intimal hyperplasia in a large bAVM vessel) and destructive (lower pictures, site of vessel wall inflammation with infiltration of mononuclear and polymorphonuclear inflammatory cells and subsequent destruction of the vessel wall) bAVM vessel remodeling. The markings A and L in the microphotographs stand for adventitia and lumen, respectively

**Fig. 5:**
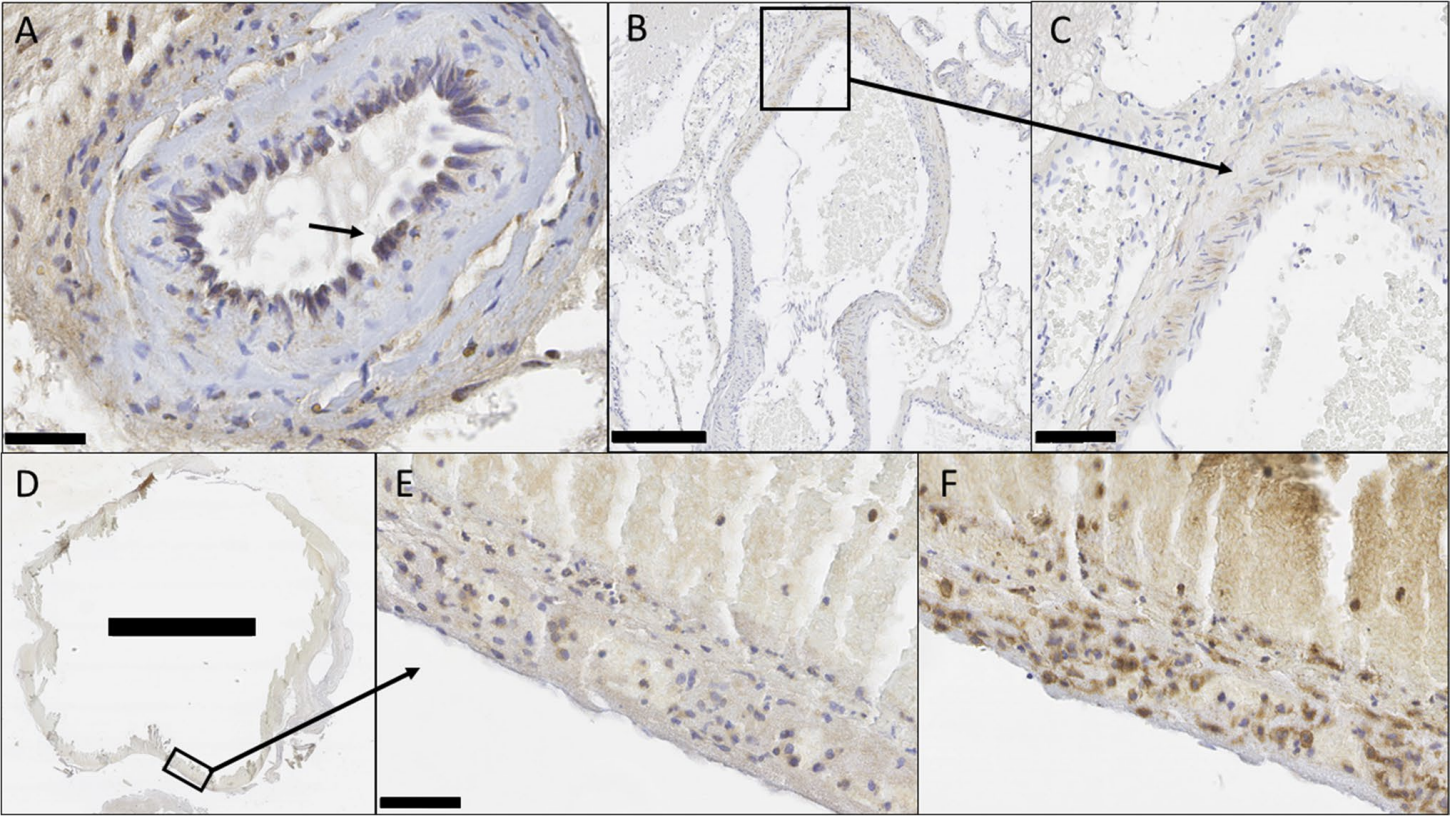
MMP9 is expressed in bAVMs. The figure shows MMP9 expression (in brown) in endothelial cells (marked by black arrows) of a bAVM vessel (**A**, scale bar 25 um). MMP9 expression was also observed in mural cells of the smooth muscle cell layer (lower magnification in (**B**) (scale bar 250 µm) and higher magnification in (**C**) (scale bar 50 µm)). Example of a large bAVM vessel with inflamed wall and MMP9 expression is presented in lower magnification in (**D**) (scale bar 2.5 mm), with a higher magnification view showing MMP9 expression (in brown) in (**E**) (scale bar 25 µm) and in inflammatory cells (CD45 in brown) in adjacent section in (**F**)

In addition to COX2, the expression of EP2 which is the receptor-mediating COX2–PGE2–NFkB signaling involved in ectatic arterial remodeling during aneurysm formation [3], was observed in the mural SMC layer, in the endothelium of small and large bAVM vessels, as well as in the inflammatory cells surrounding bAVM vessels (Fig. 3). Expression of EP2 in mural cells at regions of intimal hyperplasia in large bAVM vessels (Fig. 4), as well as in inflammatory cells infiltrating the bAVM vessel wall at regions of degenerative vessel wall remodeling (Fig. 4) suggests involvement of COX2-PGE2-NFkB signaling pathway in both adaptive and degenerative bAVM vessel remodeling. Interestingly, expression of matrix metalloproteinase-9 (MMP-9), a collagenase involved in vessel remodeling [18], was found in vessels of 100% (8/8) of a studied subset of bAVM samples. This MMP-9 expression localized in the bAVM vessels to the luminal surface (endothelium) and the mural SMC layer (Fig. 5), but it was also expressed by vessel wall infiltrating inflammatory cells at sites of degenerative vessel wall remodeling (Fig. 5).

### Inflammatory cell infiltration in bAVMs and COX2 expression

Inflammatory cells were observed in almost all bAVMs studied. No clear association between history of prior clinical hemorrhage and inflammatory cell infiltration was found (median inflammation score 3 in both unruptured and ruptured bAVMs, respectively, *p* = 0.461, Chi-square). Focal variation in the inflammatory score was observed only in 9/49 (18%) of the samples from which multiple sections at different depths were stained. Presurgical treatment did not appear to have an effect on inflammatory cell infiltration.

**Fig. 6:**
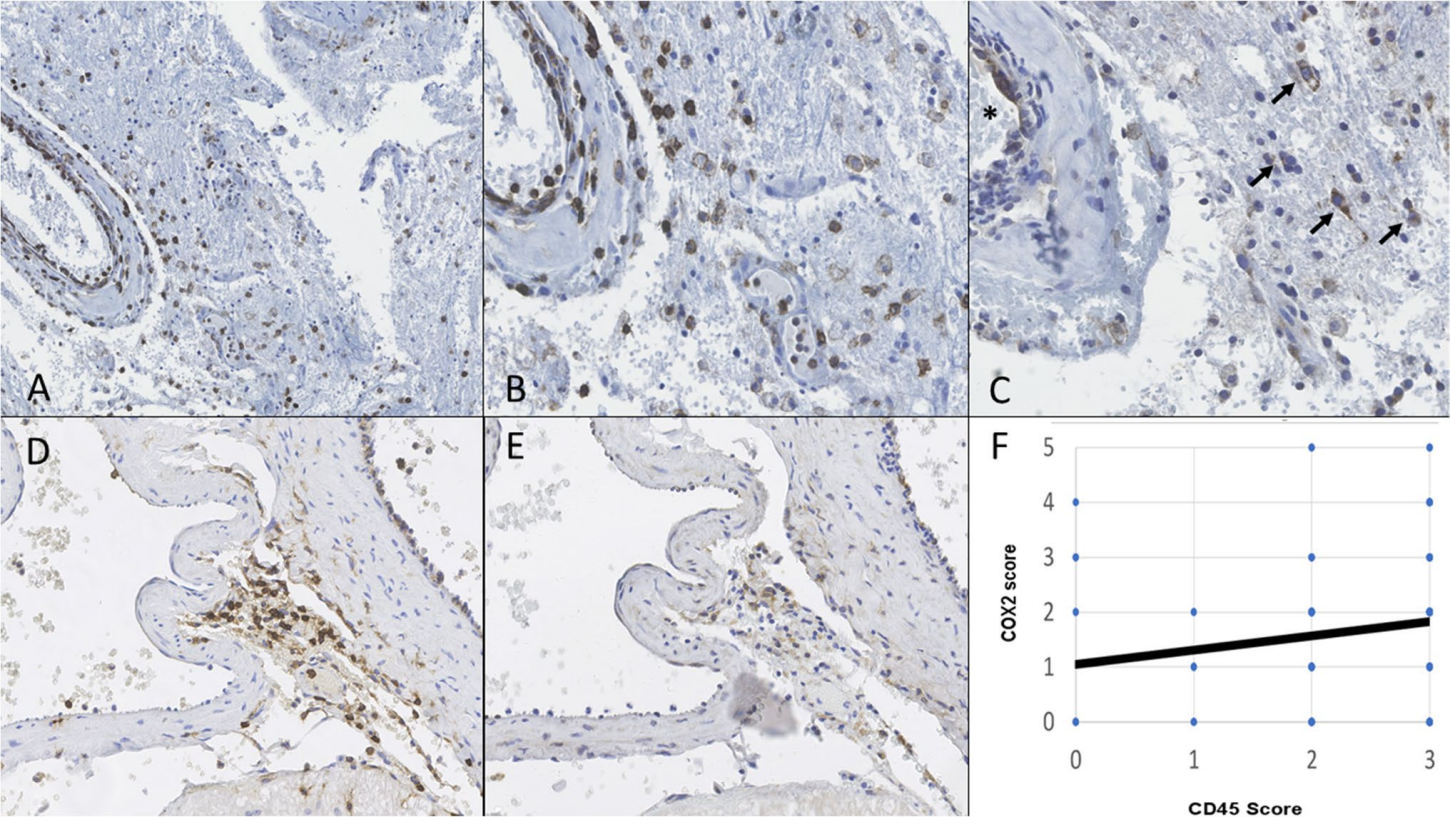
Inflammatory cells are present in bAVMs and express COX2. Figure presents in a lower (**A**) and in a higher (**B**) magnification view CD45 (panleukocyte marker, in brown) positive cells in the luminal surface, smooth muscle layer, and in the adventitia of a bAVM vessel. COX2 staining from an adjacent section (**C**) shows COX2-positive cells (in brown) at the same region as CD45 positive inflammatory cells. In C, the perivascular inflammatory cells are marked by black arrows and an asterisk (*) marks vessel lumen. Another example of co-localization of perivascular inflammatory cells (in brown, **D**) and COX2 expression (in brown, **E**) in adjacent sections is given in (**D**) and (**E**). The correlation between inflammation (*x*-axis) and COX2 expression (*y*-axis) is shown in (**F**). The following scale was used to score CD45 and COX2 immunostainings: 0 = no positive signal, 1 = 2–15 positive cells, 2 = more than 15 positive cells but < 1/3 of the surface area showing positivity, 3 = over 1/3 of the sample surface area showing positive signal

COX2 expressing inflammatory cells were observed in 76% of the samples and were found both in previously ruptured as well as in unruptured bAVMs (Tables 3 and 4). Overall inflammatory cell infiltration correlated with COX2 expression in the sample ((*r* = 0.231, *p* = 0.007, Spearman rank correlation, Tables 2, 3, and 4; Fig. 6). Furthermore, focal COX2 expression co-localized to the sites of the inflammatory cell infiltrate in adjacent sections, suggesting COX2 involvement in mediating the inflammatory reactions in bAVMs.

**Table 3:** Association of cyclo-oxygenase 2 expression with rupture status defined as hemorrhage visible in clinical radiological examination (CT or MRI)

Expression of cyclo-oxygenase 2 in immunohistochemistry	Clinical rupture status		*P* value
	*Ruptured*	*Unruptured*	
Overall in the tissue sample	76.1% (51/67)	79.7% (47/59)	.673
*Phenotype of cyclo-oxygenase 2 expressing cells*			
Endothelial cells	48.5% (32/66)	55.4% (31/56)	.472
Vascular smooth muscle cells	21.2% (14/66)	23.2% (13/56)	.829
Inflammatory cells	75.8% (50/66)	76.8% (43/56)	1.000
Parenchymal cells (glial cells and neurons)	42.4% (28/66)	37.5% (21/56)	.711

**Table 4:** Association of cyclo-oxygenase 2 expression with prior micro- or macrohemorrhage defined as presence of hemosiderin in corresponding histological sections

Expression of cyclo-oxygenase 2 in immunohistochemistry	Histological rupture		*P* value
	*Ruptured*	*Unruptured*	
Overall in the tissue sample	81.8% (63/77)	76.3% (45/59)	.522
*Phenotype of cyclo-oxygenase 2 expressing cells*			
Endothelial cells	49.3% (37/75)	54.4% (31/57)	.601
Vascular smooth muscle cells	16.0% (12/75)	24.6% (14/57)	.271
Inflammatory cells	74.7% (56/75)	78.9% (45/57)	.679
Parenchymal cells (glial cells and neurons)	41.3% (31/75)	38.6% (22/57)	.858

### COX2 expression in brain parenchyma

**Fig. 7:**
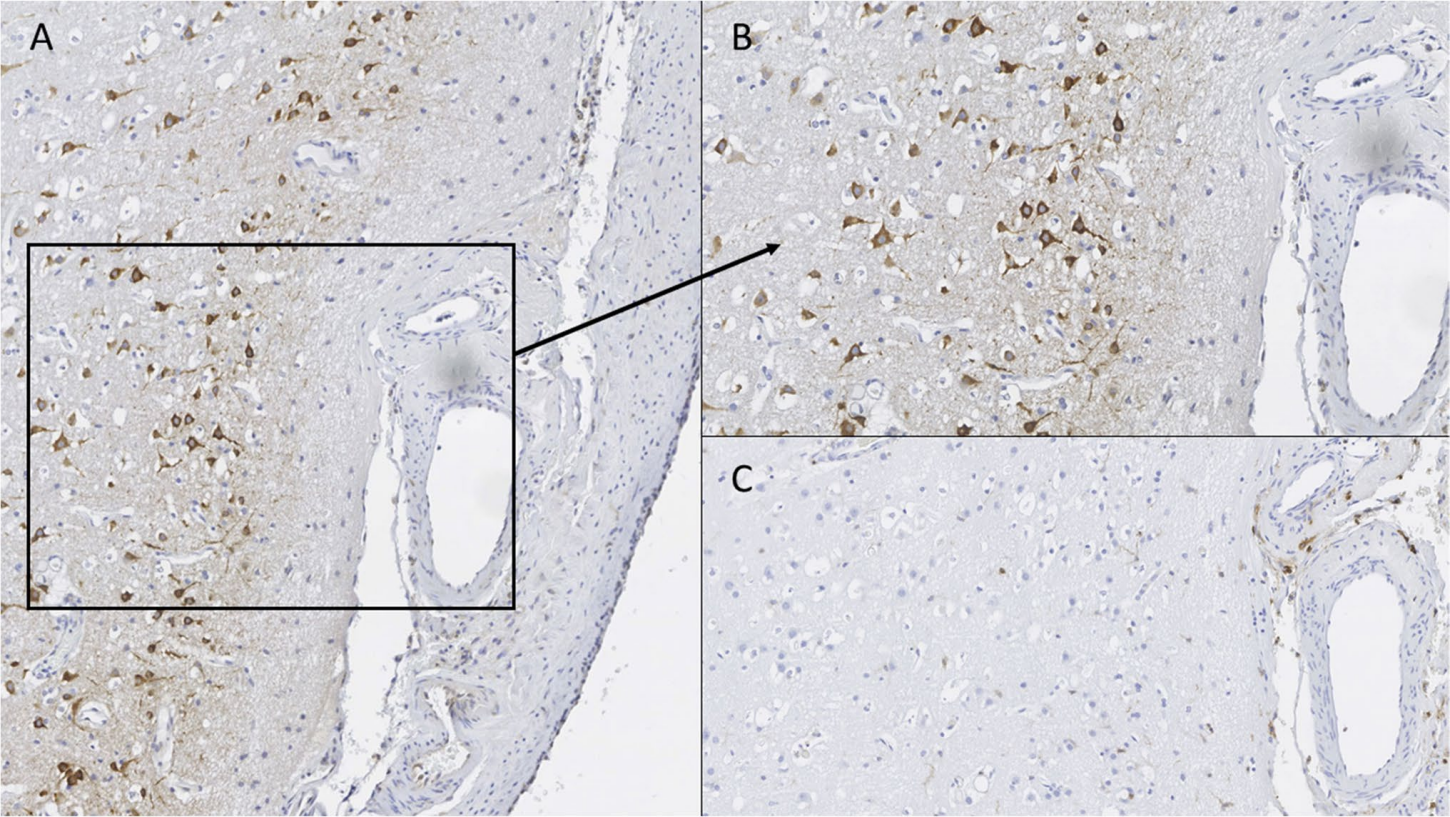
COX2 is expressed in parenchymal cells of bAVMs. The figure presents COX2 expression (in brown) in bAVM’s perinidal neural cells in a lower magnification (**A**) and in a higher magnification (**B**). The CD45 staining from adjacent section shows only few inflammatory cells (in brown) around vessels (**C**), demonstrating that the COX2-positive parenchymal cells (**A**) and (**B**) are not inflammatory cells. Morphologically, these COX2 expressing cells correspond to neurons and astrocytes COX2-positivity co-localized in adjacent sections with both GFAP-positive and GFAP-negative parenchymal cells (Supplemental data, Fig. S3)

Somewhat surprisingly, we observed COX2 expression also in the brain parenchyma either incorporating to or surrounding the bAVM nidus in 39% (53/135) of the studied samples (Fig. 7; Tables 2–4). This parenchymal COX2 expression, scored separately of the nidal vessels, associated with inflammation seen in the sample (*p* = 0.001, Chi-square). Parenchymal COX2 expression did not associate with clinical (CT or MRI confirmed) bAVM rupture or hemosiderin, which is a sign of prior micro- or macrohemorrhage in the bAVM (Tables 3 and 4). Presurgical treatments were not associated with the parenchymal COX2 expression seen in the samples. In the parenchyme, the COX2 expression co-localized with both GFAP-positive and GFAP-negative cells (Supplemental data, Fig. S3).

### COX2 expression—not a reaction to prior hemorrhage and no correlation with clinical presentation

Although our data suggests involvement of COX2 signaling in (1) remodeling of bAVM vessels, (2) inflammation of the bAVM nidus, and (3) in the pathophysiology of the adjacent brain tissue, we did not observe any significant association of COX2 expression with prior clinically diagnosed rupture (Table 3). We did not either find any association between COX2 expression and histologically defined signs of prior hemorrhage (Table 4), or symptomatic epilepsy (Table 1) that could be considered a clinical sign of hemosiderin accumulation from past microhemorrhages. Radiotherapy or embolization given prior surgical treatment did not associate with COX2 expression either.

### Multivariate modeling

In a logistic regression model including age, gender, prior embolization, prior radiosurgery, hemosiderin (histological sign of prior hemorrhage), inflammation, and COX2 expression, only female gender (OR, 2.8; 95% CI: 1.0–7.7) was significantly associated with rupture prior to surgery of the bAVM. None of these variables were independently associated with inflammation of the bAVM in a second logistic regression model that included the same variables and rupture status as explaining variables. In a third logistic regression model with COX2 expression as the outcome variable and age, gender, rupture status, prior embolization, prior radiosurgery, hemosiderin (histological sign of prior hemorrhage), and inflammation as explaining variables, only prior radiosurgery was significantly associated with COX2 expression (OR, 7.7; 95% CI: 1.2–49.5).

## Discussion

We investigated whether COX2-mediated signaling is also involved in the pathophysiology of brain AVMs (bAVM), characterized by ectatic, enlarged capillaries under abnormally high flow conditions. According to our data, COX2 expression is indeed induced in approx. 80% of bAVMs, in which it seems to be involved in the inflammatory process in bAVM vessels, nidus, and in the adjacent brain parenchyma. Moreover, our data shows that this COX2 expression is not just a reaction to prior rupture or prior treatment but instead COX2 expression is also found in unruptured and previously untreated bAVMs (Table 1), implying that COX2 signaling could be a potential target for drug therapy modulating inflammation and vessel remodeling and in bAVMs.

### COX2-mediated signaling in ectatic vessel remodeling, in aneurysm formation, and in bAVMs

COX2 expression can be triggered by abnormal blood flow [3, 4, 26, 28]. It mediates vessel dilation, as well as ectatic vessel remodeling [5, 7, 27]. Involvement of COX2 signaling has been demonstrated in the formation of abdominal aortic aneurysms (type of fusiform aneurysm), as well as in the formation of intracranial aneurysms (typically saccular aneurysms [3, 4, 31]. Given the role of COX2 as a regulator of vessel diameter and the role of COX2 in the other ectatic vascular pathologies described above. It seems that COX2 expression in the bAVM vessel wall would contribute to the growth and remodeling of these vessels. Even COX2 expression in the bAVM vessels might contribute to the formation of venous ectasias and intranidal aneurysms, typical sites from which bAVM related major intracranial hemorrhage occurs. Further studies on this are warranted.

COX2 expression also amplifies the inflammatory response through an autocrine feedback loop of COX2-PGE2-EP2-NFkB-COX2 signaling in macrophages [3, 4]. This promotes vascular ectatic remodeling in part through the expression of the collagen degrading protease matrix metalloproteinase-9 (MMP9) [3, 12]. In addition to COX2 expression, we also showed the expression of MMP9 in bAVM vessels (Fig. 5). This MMP9 expression in bAVM vessels is a sign of ongoing vessel remodeling that might be triggered through the same flow-related mechanisms as in intracranial aneurysms and possibly could explain the formation of intranidal aneurysms and draining venous ectasias that are the most common sites of bAVM rupture [16, 21]. Considering the hypothesis that COX2-PGE2-EP2-NFkB signaling might be involved in flow-induced remodeling of bAVM vessels similarly to IA formation, of note is the prior observation that NFkB is activated in bAVMs, especially in inflammatory cells and endothelial cells [6].

### COX2 as the mediator and modulator of inflammation in bAVMs

Although we did not find a clear association between COX2 expression and rupture history of the bAVM, the observation that COX2 expression is induced in bAVMs implies that COX2 plays a role in the bAVM pathobiology, which in turn translates into the clinical course of the lesion. Our results also suggest that a similar autocrine positive feedback loop through COX2-PGE2-EP2-NFkB-COX2 pathway as in IA formation [3], might amplify the inflammatory cell response in bAVMs. In our series, COX2 expression associated with inflammatory cell infiltration both focally as well as quantitatively at the level of the whole section (Fig. 6). Moreover, cells of inflammatory cell phenotype expressed COX2 and expression of EP2 receptors was also observed in perivascular inflammatory cells (Figs. 3 and 4).

In addition to bAVM vessels, inflammatory cell infiltration was observed also in the perinidal parenchyma consisting of neural and glial cells (Fig. 7). Chen et al. reported similar findings in a histological study of inflammation in bAVMs [10]. In great portion of our samples, this perinidal area surrounding bAVM structures was also shown to express COX2 (Fig. 7). It seems possible that the inflammation in the vessels of the bAVM nidus might promote production of proinflammatory cytokines also in the adjacent perinidal brain tissue and subsequently induce inflammatory cell infiltration into the parenchyma, as well as COX2 expression in parenchymal cells and perinidal inflammatory cells. Based on immunohistochemistry, it seems that not all of the COX2-positive cells in the brain parenchyma express CD45 (marker for inflammatory cells) (Fig. 7), which implies that at least in some cases, COX2 is indeed expressed by neural or glial cells. COX2 expression co-localized with both GFAP-positive and GFAP-negative cells in adjacent sections (Fig. S3), in turn suggests that these parenchymal cells include both neural and glial cells. The parenchymal COX2 expression implies that parenchymal cells might promote inflammatory cell recruitment to the bAVM nidus and raises the intriguing hypothesis that inflammation of the bAVM nidus might in fact be triggered by induction of COX2 expression in the brain parenchyme instead of or in addition to vessel inflammation causing inflammation in the parenchyme. Of note is the finding that in our multivariate analysis including both clinical and histological variables, only prior radiosurgery was significantly associated with COX2 expression. This probably reflects the known fact that radiosurgery induces inflammation, and as a consequence, the expression of proinflammatory stimuli is likely to increase as well. However, radiosurgery is not the main cause for COX2 expression in bAVMs, given that most bAVMs showing COX2 expression had not underwent radiotherapy (Table 1).

### COX2 as putative target for anti-inflammatory drug therapy stabilizing bAVMs

Our results indeed show that COX2 expression is induced in most of the bAVMs and is expressed by luminal cells, inflammatory cells, SMCs, and the brain parenchyma surrounding the bAVM nidus. Our results furthermore suggest involvement of the COX2-PGE2-EP2-NFkB signaling pathway in the remodeling of bAVM vessels, as well as in induction and amplification of the inflammatory response in bAVMs. As such, our results suggest that COX2 could be a target for drug therapy modulating inflammation and vessel remodeling in bAVMs. This possibility calls for further studies on the role that COX2 plays in the pathophysiology of bAVM development and progression, and how this translates to the clinical course of the disease, especially since drugs that inhibit COX2 are widely available in clinical practice worldwide.

Considering the prospect of using pharmaceutical COX2 inhibition to stabilize bAVMs, it is worth noting that 43% (47/109) of the COX2 expressing bAVMs had not experienced clinically evident rupture (Table 1) and most of them had not had prior radiosurgery or embolization (Table 1). Moreover, 76% (45/59) (Table 4) of the bAVMs that had no histological sign of prior clinical or subclinical rupture, nevertheless had COX2 expression. This implies that the observed COX2 expression is not a reaction to prior rupture, nor a reaction to prior treatment, although embolization and especially radiosurgery may well enhance it.

## Conclusion

We show the expression of cyclo-oxygenase 2, an inducible mediator of vessel remodeling and inflammation in the majority of bAVMs regardless of clinical presentation and rupture status. Our observations suggest that the role of cyclo-oxygenase 2 in the pathophysiology and progression of bAVMs merits further studies, as does also the possible role of cyclo-oxgenase 2 as a target for drug therapy stabilizing bAVMs.

## Supplementary information

ESM 1 (DOCX 4.91 mb)
