# Supplementary material for: Cyclo-oxygenase 2, a putative mediator of vessel remodeling, is expressed in the brain AVM vessels and associates with inflammation
Source: Acta Neurochir (Wien). 2021 Jun 29;163(9):2503–14. doi: 10.1007/s00701-021-04895-z (PMC8357659; doi:10.1007/s00701-021-04895-z)
Supplement: Supplementary file 1 — (DOCX 4.91 mb) [file 701_2021_4895_MOESM1_ESM.docx]

Data supplement

“Cyclo-oxygenase 2 is expressed in the brain AVMs and associates with inflammation – a putative mediator of vessel remodeling in brain AVMs”

by Keränen S et al.

**Figure S1. Examples of bAVMs in digital subtraction angiography.**

The exceedingly high flow caused by the arteriovenous shunting of nidal vessels in arteries supplying the bAVM or in veins draining it, can induce through flow mediated vessel remodeling the formation of aneurysm in the feeding arteries (A,B,C) or venous ectasias (D) in the draining veins of the bAVM. In addition to these manifestations of ectatic vessel remodeling, formation of aneurysms in the actual nidal vessels (E, F) of the bAVM is not uncommon. Aneurysms are marked with a black circle in A-C and E-F, while in D the venous ectasias of the draining vein are encircled. The black arrows point to the AVMs. The same AVM is shown in different phases post contrast injection in A and B. The small insert figure shows the same AVM at a different phase in E and F, and also from a different projection in E.


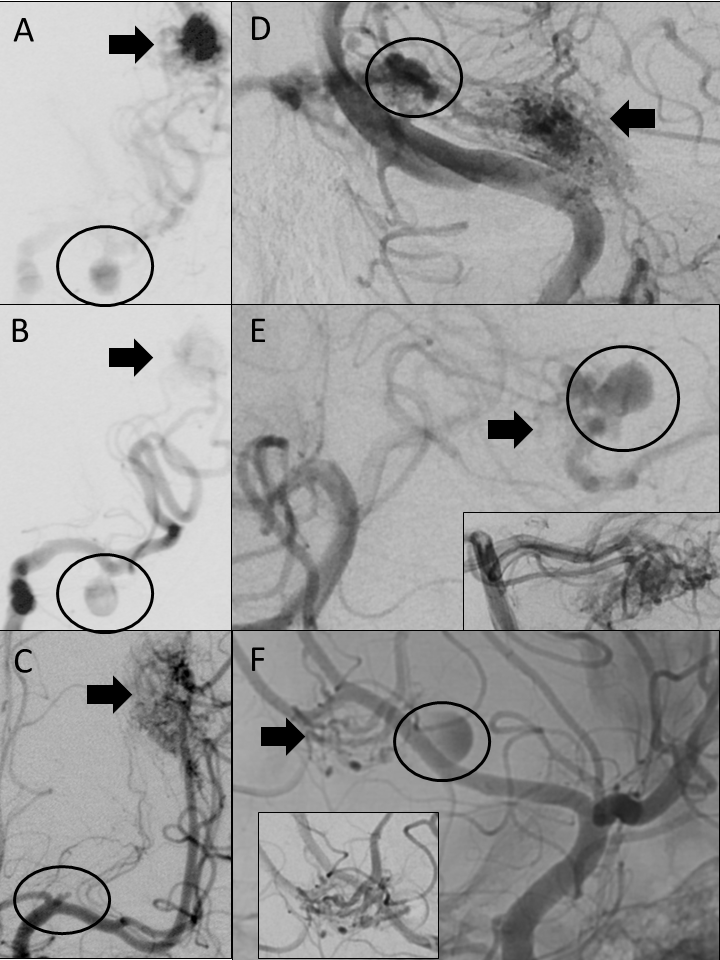


**Figure S2. Quantitative real time PCR for cyclo-oxygenase 2.**

COX2 expression was found in 4 of the 6 bAVM samples studied with RT-PCR (samples 1-6) but COX2 expression was undetermined on 6 Circle of Willis samples (7-12). A bowel sample was used as a positive control.

**Figure S3 COX2 and GFAP immunostainings from adjacent sections.**

Panel A/ COX2 and GFAP immunostainings from adjacent sections demonstrate how COX2 expressing cells in the brain parenchyma (in A and with higher magnification in B) co-localize with GFAP expression (in C and with higher magnification in D), suggesting that glial cells express COX2.


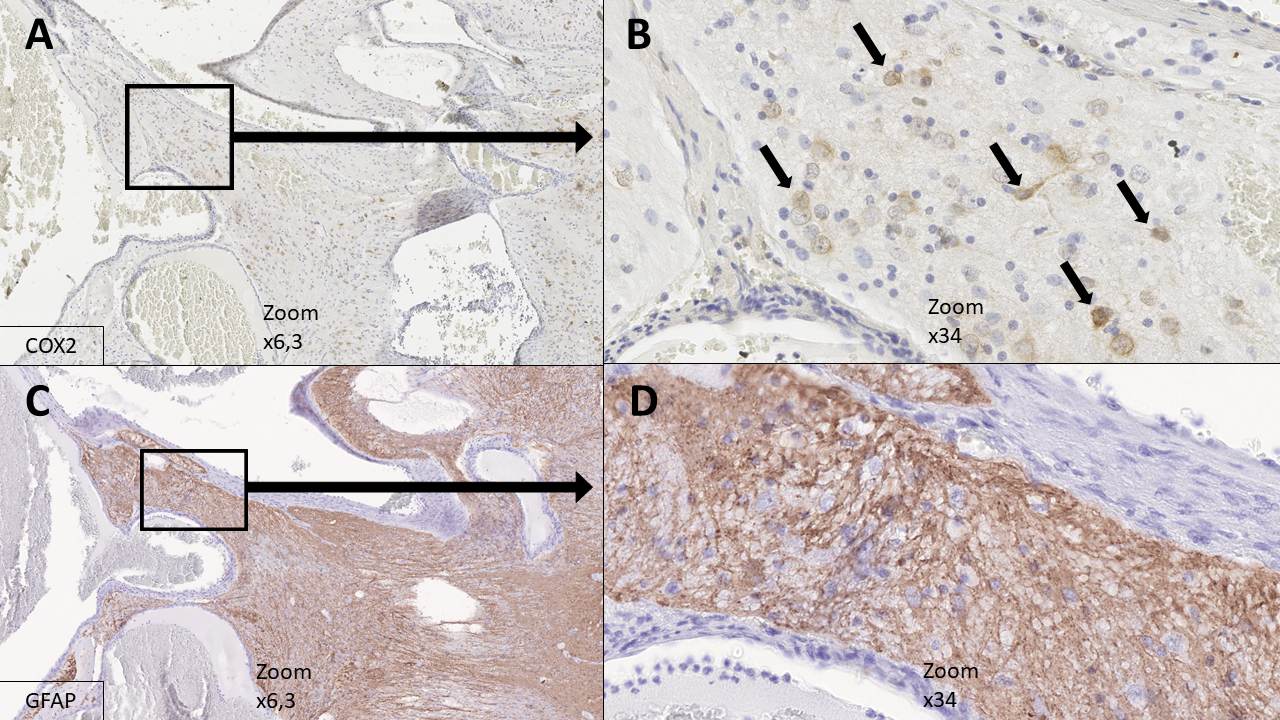


**Panel B/ Also COX2 expressing (in A and with higher magnification in B) but GFAP negative parenchymal cells (in C and with higher magnification in D) with a glial or neuronal morphology were found, suggesting that some of the parenchymal cells expressing COX2 were not glial cells but neurons.**


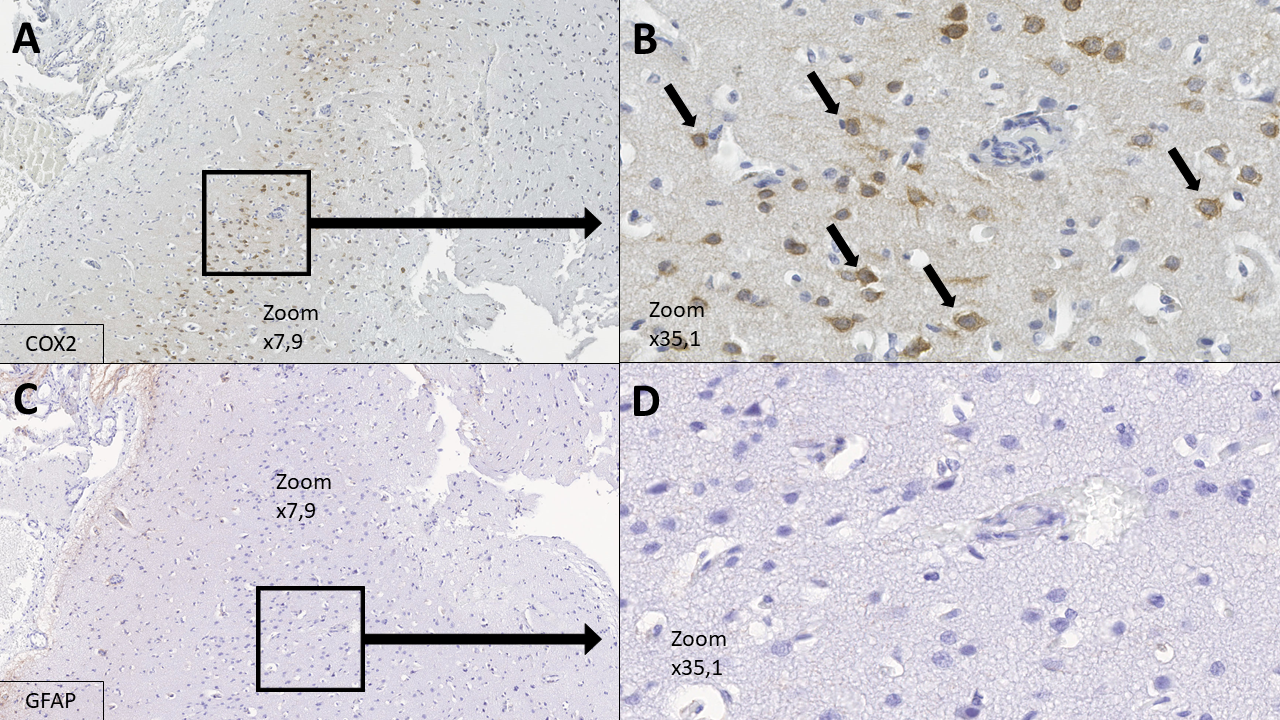


Figure S4. A schematic presentation of role of COX2 – derived prostaglandins and other paracrine hormones in the regulation of vascular tone and remodeling [5,21,32,35,37]


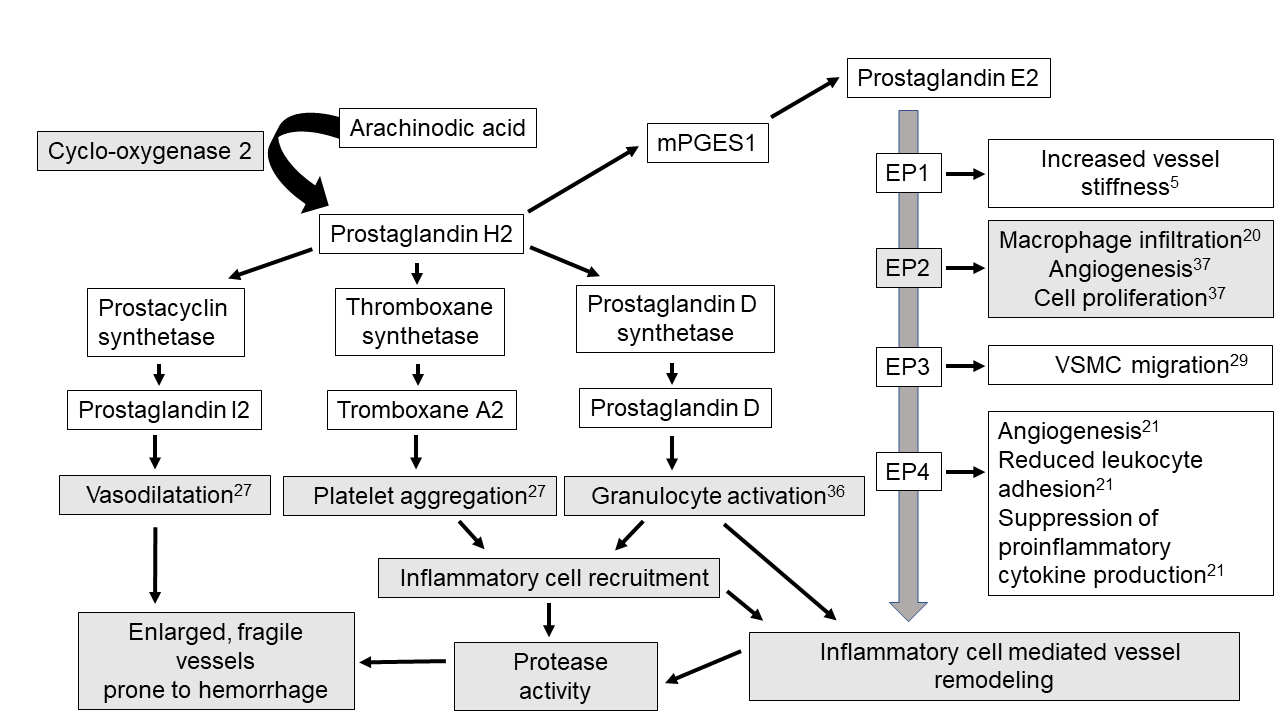


**Methods**

Isolation of RNA and RT- PCR

Paraformaldehyde-fixed (4 %) and paraffin-embedded tissue samples were cut in a microtome and deparaffinized. RNA isolation was performed by using the Recover All Total Nucleic Acid Isolation Kit for FFPE (ThermoFisher Scientific, Waltham, Massachusetts) according to the instructions of the manufacturer. The RNA was cleaned by using RNA Clean and Concentrator-5 (Zymo Research, CA, USA). Total RNA was reverse transcribed into cDNA using random hexamers and RevertAid Reverse Transcriptase (Thermo Fisher Scientific). For RT-PCR, PowerUp SYBR Green master mix (ThermoFisher Scientific, Waltham, Massachusetts) was used. Primers for COX2 are given below. Expression of GAPDH was used as an endogenous control for the RT-PCR.

1. **COX2**

|  | **Start** | **Stop** | **Length** | **Tm** | **GC%** |
| --- | --- | --- | --- | --- | --- |
| Forward  **TACTGGAAGCCAAGCACTTT (Sense)** | 1588 | 1608 | 20 | 62 | 45 |
| Reverse  **GGACAGCCCTTCACGTTATT (AntiSense)** | 1666 | 1686 | 20 | 62 | 50 |

1. **GAPDH**

| Forward  **GCAAGAGCACAAGAGGAAGA (Sense)** | 1107 | 1127 | 20 | 62 | 50 |
| --- | --- | --- | --- | --- | --- |
| Reverse  **CTACATGGCAACTGTGAGGAG (AntiSense)** | 1189 |  |  |  |  |

Immunohistochemistry

Paraformaldehyde-fixed (4 %) and paraffin-embedded 4 µm thick tissue sections were deparaffinized, rehydrated and stained with immunohistochemistry. An antigen retrieval was performed by heating the sections 2x5min close to boiling temperature in 0.1 M pH6 citrate buffer (ThermoFisher Scientific, Waltham, Massachusetts). After cooling down in citrate buffer and a PBS rinse, serum block was performed using 3 % normal horse serum (Biowest, Nuaillé, France) in PBS for 30min in room temperature. This was followed by overnight incubation at +4°C with the primary antibody diluted 1:100 in 1.5% normal horse serum in PBS. Used primary antibodies were mouse monoclonal anti-COX2 (clone CX-294, DAKO, Glostrup, Denmark), mouse monoclonal anti-CD45 (clones 2B11+PD7/26, DAKO), rabbit polyclonal anti-EP2 (LifeSpan BioSciences, Seattle, Washington), mouse monoclonal anti-MMP9 (clone 56-2A4, LifeSpan BioSciences), mouse monoclonal anti-CD31 (clone JC70A, DAKO) and mouse monoclonal alfa-SMA (clone 1A4, DAKO). After the incubation with the primary antibody and a 3x5 min PBS wash, the sections were incubated with a secondary antibody (dilution 1:200, Vector Vectastain kit, Vector Laboratories, Burlingame, CA) chosen according to the species where the primaries were produced) for 30 min in room temperature, followed by another 3x5 min PBS wash, a 20 min endogenous peroxidase block in 3 % H_2_O_2_ (ThermoFisher Scientific) in PBS, a 3x5min PBS wash, and finally detection of the bound biotinylated secondary antibody using the avidin-biotin conjugated horseradish peroxidase (Vector Laboratories) and DAB (3’-5’-diaminobenzidine) (Vector Laboratories) as peroxidase substrate. Hematoxylin (Vector Laboratories) was used for counterstaining.

To confirm the phenotype of COX2 expressing cells, immunofluorescence double stainings were performed for selected samples. General protocol was the same as described above, except that 3 % normal goat serum (Vector Laboratories) in PBS was used for serum block and Alexa Fluor 488 and 594 conjugated secondary antibodies (dilution 1:50 or 1:200, chosen according to species where primary antibodies were produced, Thermo Fisher Scientific, Waltham, MA, USA) for signal detection. Vectashield medium with DAPI (Vector Laboratories) was used for mounting. LSM800 Zeiss confocal microscope system (Carl Zeiss Ag, Oberkochen, Germany) was used for immunofluorescence imaging with 405/488/555nm diode lasers and appropriate emission filters (10x/0.3 or 20x/0.5 PlanApo objectives, 512x512 and 1024x1024 frame sizes). Image processing was performed by ImageJ (Rasband, W.S., National Institutes of Health, Bethesda, Maryland).

Histological analysis

Immunoperoxidase stained tissue sections were scanned with Hamamatsu digital slide scanner (Hamamatsu Photonics K.K., Naka-ku, Hamamatsu, Shizuoka, Japan), following histological analysis using NDP.view2 viewing software (Hamamatsu Photonics K.K.). The presence and extend of positive immunostaining for COX2 or CD45 were scored according to the following scale: 0= no positive signal, 1= 2-15 positive cells, 2= > 15 positive cells but < 1/3 of the surface area positive, 3= > 1/3 of the sample surface area showing positive signal. The presence of hemosiderin observable in the hematoxylin background was scored using the same scale. The samples were scored by observers (S.K and J.F. independently, and to determine interobserver variability 76 of the samples were scored by both observers. Primary agreement on COX2 score was attained in 68,8% of the samples evaluated by observers individually. After a thorough analysis done jointly on samples where no consensus was primarily obtained, agreement on COX2 score was reached in all samples. To examine focal variation in COX2 expression and inflammation, we stained multiple sections obtained from different tissue depths of the same bAVM sample from 55 samples.

**References**

20. Jackson ZS, Gotlieb AI, Langille BL (2002) Wall tissue remodeling regulates longitudinal tension in arteries. Circ Res 90:918–925

21. Jandl K, Stacher E, Bálint Z, Sturm EM, Maric J, Peinhaupt M, Luschnig P, Aringer I, Fauland A, Konya V, Dahlen SE, Wheelock CE, Kratky D, Olschewski A, Marsche G, Schuligoi R, Heinemann A (2016) Activated prostaglandin D2 receptors on macrophages enhance neutrophil recruitment into the lung. J Allergy Clin Immunol 137:833–843

32. Seo MJ, Oh DK (2017) Prostaglandin synthases: molecular characterization and involvement in prostaglandin biosynthesis. Prog Lipid Res 66:50–68

35. Yokoyama U, Iwatsubo K, Umemura M, Fujita T, Ishikawa Y (2013) The prostanoid EP4 receptor and its signaling pathway. Pharmacol Rev 65:1010–1052

36. Zhang J, Zou F, Tang J, Zhang Q, Gong Y, Wang Q, Shen Y, Xiong L, Breyer RM, Lazarus M, Funk CD, Yu Y (2013) Cyclooxygenase-2-derived prostaglandin E2 promotes injury-induced vascular neointimal hyperplasia through the E-prostanoid 3 receptor. Circ Res 113:104–114

37. Zhao L, Wu Y, Xu Z, Wang H, Zhao Z, Li Y, Yang P, Wei X (2012) Involvement of COX-2/PGE2 signalling in hypoxia-induced angiogenic response in endothelial cells. J Cell Mol Med 16:1840–1855
